# Plant-Derived Substances with Antibacterial, Antioxidant, and Flavoring Potential to Formulate Oral Health Care Products

**DOI:** 10.3390/biomedicines9111669

**Published:** 2021-11-11

**Authors:** Marco A. Lugo-Flores, Karen P. Quintero-Cabello, Patricia Palafox-Rivera, Brenda A. Silva-Espinoza, Manuel Reynaldo Cruz-Valenzuela, Luis Alberto Ortega-Ramirez, Gustavo Adolfo Gonzalez-Aguilar, Jesus Fernando Ayala-Zavala

**Affiliations:** 1 Centro de Investigacion en Alimentacion y Desarrollo, A.C., Carretera Gustavo Enrique Astiazaran Rosas, No. 46, Col. La Victoria, Hermosillo C.P. 83304, Sonora, Mexico; marco.lugo.mc18@estudiantes.ciad.mx (M.A.L.-F.); karen.quintero.mc18@estudiantes.ciad.mx (K.P.Q.-C.); patricia.palafox.mc18@estudiantes.ciad.mx (P.P.-R.); bsilva@ciad.mx (B.A.S.-E.); reynaldo@ciad.mx (M.R.C.-V.); gustavo@ciad.mx (G.A.G.-A.); 2 Unidad Académica San Luis Río Colorado, Universidad Estatal de Sonora, Carretera, Sonoyta-San Luis Río Colorado km. 6.5, Parque Industrial, San Luis Río Colorado C.P. 83500, Sonora, Mexico; luis.ortega@ues.mx

**Keywords:** bacterial biofilm, oral microbiota, free radicals, synergism, plant extract

## Abstract

Bacterial diseases and reactive oxygen species can cause dental caries and oral cancer. Therefore, the present review analyzes and discusses the antibacterial and antioxidant properties of synthetic and plant-derived substances and their current and future patents to formulate dental products. The reviewed evidence indicates that chlorhexidine, fluorides, and hydrogen peroxide have adverse effects on the sensory acceptability of oral care products. As an alternative, plant-derived substances have antimicrobial and antioxidant properties that can be used in their formulation. Also, adding plant metabolites favors the sensory acceptability of dental products compared with synthetic compounds. Therefore, plant-derived substances have antibacterial, antioxidant, and flavoring activity with the potential to be used in the formulation of toothpaste, mouth rinses, dentures cleansers-fixatives, and saliva substitutes.

## 1. Introduction

Oral diseases are a significant health burden for many countries, causing pain, discomfort, deformity, and even death; it is estimated that these affect nearly 3.5 billion people [1]. According to the Global Burden of Disease, untreated dental caries in permanent teeth is the most common health condition [2]. Severe periodontal disease may result in tooth loss, affecting almost 10% of the global population. Also, oral cancer (lip or mouth cancer) is one of the three most common cancers in Asia [3,4]. Treatment for oral health conditions is expensive and usually not part of universal health coverage. In most high-income countries, dental treatment costs are almost 5% of the total health expenditure [5]. Most low- and middle-income countries are unable to provide services for preventing and treating oral health conditions. In addition, inadequate hygienic procedures, high sugar diets, tobacco use, and alcohol are factors contributing to oral diseases [6]. Most oral health conditions are mostly preventable and can be treated in their early stages.

*Streptococcus mutans* is among the main bacteria responsible for dental diseases and their complications; therefore, it is important to understand the mechanisms involved in its pathogenicity. Increasing cell density and nutrient deficiency promotes *S. mutans*’ growth by expressing virulence factors, including biofilm development [7,8]. Biofilms are bacterial communities embedded in an extracellular polymer matrix consisting of polysaccharides, proteins, and DNA capable of generating a suitable microenvironment for bacterial development, protecting them from toxic agents, and assuring survival during environmental stresses [8]. In the particular case of *S. mutans*, it synthesizes glucans as polymeric substances using glucosyltransferases that metabolize sucrose [9]. Other products of sugar metabolism are acetic, formic, and lactic acids propitiating a suitable medium for bacterial growth and affecting dental surfaces [10]. Therefore, controlling harmful bacteria and oxidative stress in the oro-dental area is a severe concern for global health authorities.

Some synthetic substances, such as chlorhexidine and fluorides, among other compounds, have antibacterial activity against *S. mutans*; therefore, they have been incorporated into different dental products, such as pastes and mouthwashes [11]. However, these compounds can cause adverse effects, such as dental pigmentation, changes in taste perception, and burning sensation that cause their rejection by consumers [12,13]. Plant extracts are the current trend in the market, but it is also essential to consider their antibacterial, antioxidant, and sensory characteristics. Plant essential oils are constituted by secondary metabolites, such as terpenes, phenols, and aldehydes with antibacterial, antioxidant, and flavoring properties [14]. Other industries take advantage of the essential oil properties; some examples of their applications are found in the food, pharmaceutical, and cosmetic industries. In addition, these plant extracts have been useful for the in vitro inactivation of *Escherichia coli*, *Staphylococcus aureus*, *Listeria monocytogenes,* and *S. mutans*. Therefore, the incorporation of essential oils into oral products can be found in mouthwashes and toothpaste.

Essential oils have antioxidant and anticarcinogenic properties that could be useful to preserve oral health. For example, essential oils of *Thymus serpyllum*, *Mentha piperita*, *Juniperus communis, Rosmarinus officinalis, Melissa officinalis, Achillea millefolium, Zingiber officinale*, and *Helichrysum arenarium* inhibited the proliferation of oral squamous cell carcinoma culture and SCC-25 cell line [15]. Besides, *Menta piperita* extracts at 1.5 mg/mL showed a cytotoxic effect on tumor cell lines. At the same time, rosmarinic and carnosic acids from *Melissa officinalis* and rosemary essential oils can inhibit growth and stimulate apoptosis in cancer cells at the same dose [15]. This evidence highlights that plant essential oils are a source of antioxidant agents with potential anticarcinogenic activity.

The search for substances to preserve oral health showed that synthetic compounds, such as chlorhexidine cause irritation, dryness, and alteration in flavors’ perception [16]. As a counterpart, essential oils can provide pleasant sensory attributes; for example, *Cymbopogon citratus* or lemongrass essential oil has a fresh citrus smell and a sweet-spicy flavor [14]. On the other hand, essential oils obtained from *Melaleuca bracteata* and *Ocimum basilicum* plants contain gallic acid and quercetin that produce sweet and bitter flavors, respectively [17]. Therefore, the substance election is important for formulating dental products with antibacterial activity, antioxidant capacity, and sensory properties. Thus, the analyzed evidence indicates that the use of plant extracts shows a broad profile of advantages (antibacterial, antioxidant, and flavoring properties) to formulate oral health care products compared to conventionally used synthetic compounds.

## 2. Eubiosis vs. Dysbiosis of Oral Microbiota: Health Impact

The oral cavity favors the development of many microorganisms; this area includes epithelial surfaces of mucous membranes and dental pieces prone to be invaded mainly by bacteria [18]. Facultative anaerobes are colonizers of oral surfaces, mainly of the *Streptococcal* genus, found in the subgingival and dental areas forming biofilms and plaque [18]. It is currently estimated that more than 700 bacterial species co-exist in oral tissues; some of them can present benefits to the host, while others can be harmful [19].

The oral microbiota comprises many microorganisms, including viruses, bacteria, Archae, fungi, and protozoa; these participate in the homeostasis and maintenance of oral health. Microbiota must co-exist in harmony among them and with the host to contribute to their well-being; this balance and harmony is called eubiosis, while the opposite state is named dysbiosis. Oral eubiosis can prevent the colonization of exogenous microorganisms because they are better adapted to these surfaces [20]. However, the increment in certain pathogens can be detected when oral dysbiosis occurs. Among the most indicated oral pathogenic bacterial species are *S. mutans* [21]*;* while other streptococci can be considered benefic bacteria, e.g., *S. sanguinis*, *S. oralis*, *S. gordonii,* and *S. mitis* produced hydrogen peroxide and extracellular proteases that inhibited the growth of pathogenic bacteria in the oral environment [22]; however, hydrogen peroxide also causes oxidation [23]. It has to be considered that many other non-cultivable bacteria could be involved in the symptomatology of dental caries.

**Figure 1:**
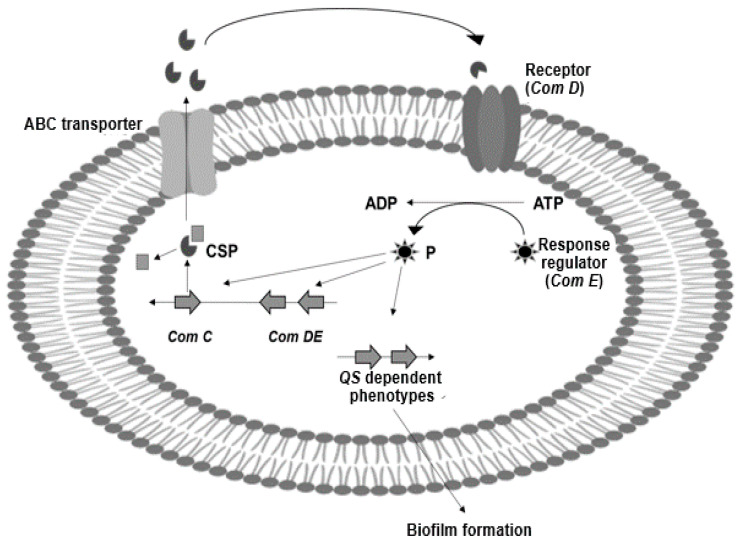
The intercellular communication system of *S. mutans*. Planktonic cells initiate intercellular communication through the excretion of a stimulating peptide (CSP), which is recognized and activates the signaling cascade to express genes involved in biofilm formation.

Using *S. mutans* as a bacterial model to explain its pathogenicity caused during dysbiosis, Figure 1 shows how this bacterium activates *quorum sensing* to control biofilm production and other virulence factors [7,8]. ComCDE secretes a competent stimulating peptide (CSP), acting also as a signal molecule; ComC synthesizes this peptide, and it is recognized and phosphorylated by the transmembrane histidine kinase (ComD) and its response regulator (ComE) [8]. The phosphorylated peptide activates the signaling cascade, expressing different genes involved to synthesize proteins and enzymes that *S. mutans* use to adhere to dental surfaces and form biofilms. 

*S. mutans* can adhere to dental surfaces by using two different mechanisms for producing polymeric substances [9]. The first mechanism is characterized by the presence of adhesins, such as the antigen P (SpaP) and protein A associated with the bacterial membrane (WapA) [24]. An anchorage of the adhesins to the bacterium’s surface is required from a transpeptidase sortase A responsible for recognizing the LPXTTG motif of adhesins and breaking the peptide bond after threonine [8]. The cleavage of the peptide bond generates a carboxyl end of threonine, which allows the binding of the protein to the surface of the lipid II through a pentaglycine bridge, while lipid II acts to bind the peptidoglycan in the cell wall [8,9,24]. Once the bacterium is fixed to the surface, the second mechanism synthesizes extracellular polysaccharides utilizing the sucrose-dependent system.

**Figure 2:**
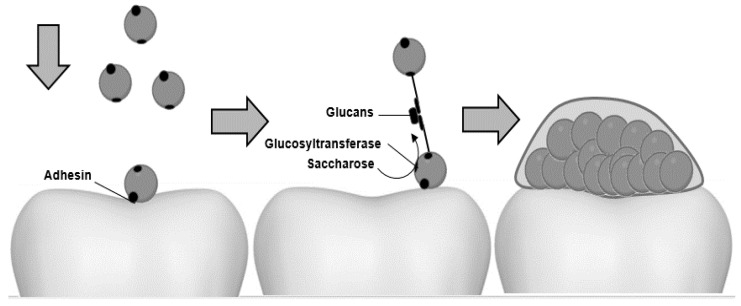
Adhesion and biofilm formation of *S. mutans* to dental surfaces. The invasion of planktonic cells to the teeth is carried out through membrane adhesins, and they use sucrose to promote cell-cell union and synthesize the extracellular polymeric matrix on dental enamel.

The sucrose availability in the oral cavity allows *S. mutans* to use an adhesion system dependent on this disaccharide. Glycosyltransferases (GTFB, GTFC, GTFD) can use sucrose as a substrate to produce insoluble glycans (α-1,3 glycosidic) and soluble glycans (α-1,6 glycosidic) [7]. Glycosyltransferases also use their carboxyl-terminal domain to bind glycan-binding proteins (GbpA, GbpB, GbpC, GbpD) found in the bacterial cell wall, allowing cell-cell adhesion [7,24]. This process produces cells and glucans accumulation, generating biofilms on dental surfaces (Figure 2) [9]. In addition, *S. mutans* metabolizes sucrose and produces organic acids capable of damaging the tooth surface [10]. Therefore, sucrose plays a vital role in *S. mutans* biofilms and damages the tooth surface through the generated by-products.

During dental caries development, degradation of the teeth’s surface occurs by demineralization of the enamel, mainly caused by acids (lactic, propionic, and formic) produced by *S. mutans*; these are introduced through the pores of the enamel and begin its degradation [10]. Caries can also cause pain, phonation problems, dental aesthetics, and even be an economic problem due to expenses in treatment and absence from work [25]. Dental caries are also a risk factor for developing endocarditis, atherosclerosis, and cerebrovascular diseases [25,26]. The relationship between periodontitis and cerebrovascular health has been evidenced by the damage of endothelial cells caused by oral inflammations and injuries to the blood-brain barrier [27,28]. On the other hand, some oral pathogenic species, such as *S. mutans,* can spread through the blood and lodge in vascular lesions, causing endocarditis [27]. Therefore, the negative health impact of pathogenic bacteria during dysbiosis stimulates the development of oral health care products with eubiotic properties.

## 3. Oral Cancer and Its Relationship with Reactive Oxygen Species

Oral cancer can be caused by spontaneous mutations and external environmental factors that induce the inadequate functioning of processes, such as cell division and the expression of defective genes [29]. As a result, this disease causes abnormal cell proliferation, tumor mass formation and decreases nutrient availability for healthy tissues [29]. These cells can also travel through the lymphatic chain and lodge in other organs and tissues, such as the mouth, causing malignant neoplasms that damage the oral cavity [30]. The oral squamous cell carcinoma is the primary tumor responsible for developing oral cancer, capable of producing 90% of all malignant neoplasms [31]. This type of cancer is the sixth most common worldwide, affecting 354,864 people and causing 177,384 deaths in 2018 [3,4]. Therefore, it is important to understand the development of this pathology since it can cause irreversible damage to oral tissues and death.

Dysbiosis of the oral microbiota can also cause cancer through different mechanisms of action. The first mechanism involves stimulating chronic inflammation caused by microorganisms, such as *Fusobacterium nucleatum* and *Porphiromonas gingivalis* [32]. These bacteria generate periodontal diseases that stimulate the production of inflammatory mediators, damaging fibroblasts, epithelial, and endothelial cells [33]. Some examples of these mediators are NF-kB, STAT3, and H1F-α that trigger genetic mutations, leading to cancer development [32]. On the other hand, it has been reported that the increase in IL-1β favors the production of angiogenic factors, such as TNF-α (tumor necrosis factor), which is responsible for increasing the content of malignant tumors in oral tissues [33]. 

**Figure 3:**
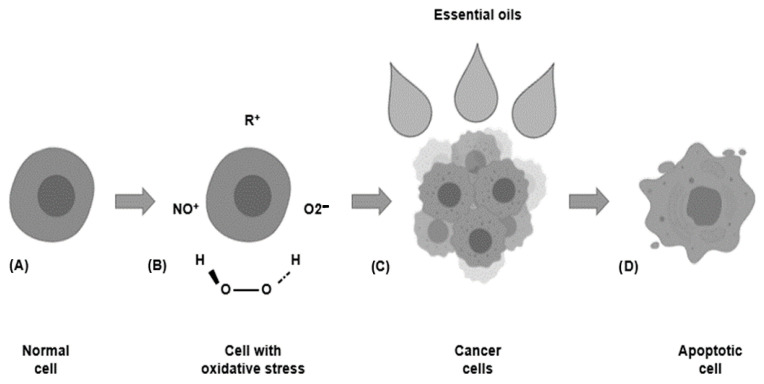
(**A**) Healthy cells constituting oral tissues; (**B**) Oncogenic metabolites stimulate oxidative stress and produce cancer cells. (**C**) Essential oil constituents interact with carcinogenic cell lines, and (**D**) promote their apoptosis.

Microbial metabolism in the oral cavity can include the synthesis of carcinogenic substances. The oral microbiota can synthesize substances related to cancer development, such as free radicals and reactive oxygen species (ROS) [33,34]. Specifically, hydrogen peroxide is a type of ROS produced by some bacteria, such as *Lactobacillus acidophilus*, *Bifidobacterium adolescentis*, and *Streptococcus* species; it increases the expression and accumulation of NADPH oxidase [33,34]. This compound can enter and damage the mitochondria, and it causes the cells’ inadequate functioning (Figure 3) [33]. Furthermore, it has been reported that ROS generate genetic damage by reducing the expression of pro-tumor genes, allowing for the presence of this type of abnormal agglomeration. Besides, hydrogen peroxide can react with Fe^2+^ producing substances that stimulate DNA mutations and cause cell cycle disorders [34]. 

The oxidative stress in the mouth is also attributed to free radicals synthesized by oral bacteria; e.g., nitric oxide (NO) is produced from nitrate in human saliva [35]. Furthermore, it has been reported that the increase of free radicals in the environment causes damage to cellular structures and promotes mutations related to cardiovascular disease and cancer [36]. Also, there is a connection between periodontal diseases produced by oral bacteria and the content of carcinogenic substances. This effect was attributed to the high amounts of free radicals produced by the host’s immune system in the presence of oral bacterial infection [33,36]. Therefore, oral microbiota and ROS control can be considered a target to prevent oral cancer and caries.

## 4. Conventional and Alternative Ingredients with Antibacterial, Antioxidants, and Flavoring Properties Used in Dental Health Care Products

### 4.1. Antibacterial Properties

The addition of antibacterial compounds in toothpaste and mouth rinses can help to eliminate harmful bacteria. Toothpaste comprises different ingredients for oral cleansing, such as fluorides and abrasives [37]. At the same time, oral rinses are aqueous solutions with active components that allow oral health; both products serve as vehicles for antibacterial agents [11,38]. 

Synthetic antibacterial agents in dental products can improve oral health by inhibiting bacterial viability or reducing adhesion [39]. Chlorhexidine is one of the most used ingredients in the formulation of mouthwashes due to its ability to eradicate *S. mutans* [40]. This compound is a broad-spectrum cationic antiseptic, and at low inhibitory (MIC) and bactericidal (MBC) concentrations (MIC: 0.0094 mg/mL, MBC: 0.0094 mg/mL), it damages the cell membrane and the metabolism of cariogenic *Streptococcus*. Chlorhexidine is mixed with the saliva covering the teeth, preventing pathogenic bacteria’s adhesion and forming their biofilms [11]. Besides, this substance can bind to the surface of the teeth’ hydroxyapatite, staying there for long periods, generating a more prolonged antibacterial effect [11]. However, this capacity can be a disadvantage since chlorhexidine interaction with the tooth surface can cause pigmentation, burning, and dryness [41].

Fluorides are another chemical substance with beneficial oral health effects and are used in dental products. These compounds bind to tin ions, affecting vital enzymes in Gram-positive bacteria, adhesion processes, and biofilm formation [42]. Specifically, it has been proven that applying povidone-iodine (10%) and sodium fluoride during one week reduces the appearance of bacterial biofilms and dental plaque accumulation [43]. Another benefit of fluorides is their ability to remineralize teeth, as in the case of tin fluoride, which interacts with dental enamel forming tin fluorophosphate, which covers and protects the tooth’s surface. However, tooth exposure to these substances can generate dental fluorosis, caused by discoloration of the teeth exposed to fluoride [12]. 

Table 1 shows the advantages and disadvantages of some of the most common antibacterial compounds used in commercial mouthwashes. The listed agents include chlorhexidine, fluorides, hydrogen peroxide, hypochlorous acid, and cetylpyridinium chloride, and essential oils, which have been shown to have beneficial effects for preserving oral health by inhibiting bacterial activity [13,44]. However, these components have some disadvantages, such as dental sensitivity, teeth staining, and lability; hence their use in dental products has decreased [13,38,44]. Therefore, the search for antibacterial agents has been directed to natural derivatives, such as plant essential oils that reduce dental plaque without generating adverse effects (Table 1) [38].

**Figure 4:**
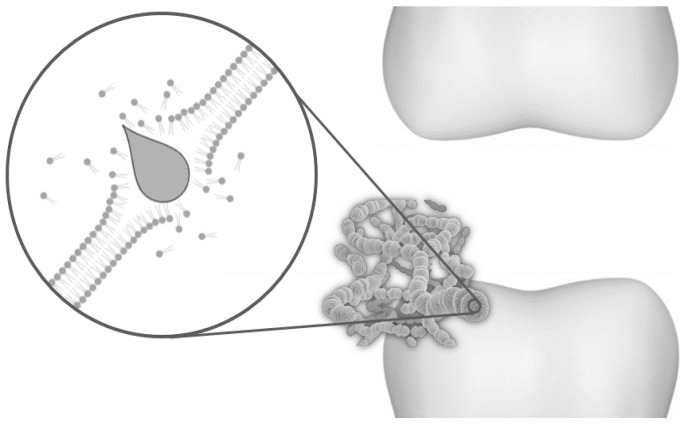
Hydrophobic molecules of the essential oils disintegrate the cytoplasmic membrane of *S. mutans*, causing the leakage of the cellular content and death.

Plant essential oils have antibacterial properties, so they have been used as substitutes for synthetic substances in oral care products. More than 3000 essential oils are currently known, of which only 300 are used in different sectors of the trade, either in food, cosmetics, and the pharmaceutical industry [45]. These are volatile aromatic liquids extracted from plant tissue consisting of a great variety of compounds. Their composition can include aldehydes, ketones, esters, phenols, alcohols, terpenes, and terpenoids [46]. The essential oils’ antibacterial activity is attributed to their ability to penetrate cell outer membranes, causing changes in fatty acid profiles, making them more permeable, and releasing cellular content (Figure 4) [47]. Another mechanism of these compounds after penetrating the cell is attacking organelles [47]. Phenolic terpenes are recognized as the active antibacterial constituents of some essential oils, as they have been proved to be effective against various microorganisms. However, there is little information related to their antibacterial effect against oral microbiota.

Thymol, menthol, eucalyptol, and methyl salicylate are the constituents of several plant essential oils with antibacterial activity against cariogenic strains of *S. mutans* (Table 2). They have been used to formulate Listerine^®^ due to their ability to inactivate *S. mutans* biofilms at low concentrations [45]. Plant compounds found in this mouthwash are menthol 0.042%, eucalyptol 0.092%, methyl salicylate 0.06%, and thymol 0.064% [48]. Other extracts with this property are *C. citratus* and *Cinnamon zeylanicum,* constituted mainly by terpenes and effective against planktonic *S. mutans*. Similarly, *Acacia nilotica*, peel extract of *Citrus aurantifolia*, and the fruit extract from *Berberis vulgaris* were also effective against this bacterium. In addition, evidence is emerging about the eubiotic effect of using plant essential oils in animal systems, e.g., the administration of thymol, eugenol, and piperine was added as an additive in chickens’ diets, resulting in the maintenance of lactobacilli and other beneficial species and reducing pathogenic bacteria in broiler chicks [49]. Similarly, the inclusion of thymol, eugenol, and piperine in the diet of nursery pigs increased the Bacteroides and decreased *Prevotella*, creating a eubiotic effect [50]. However, it must be considered that even when synthetic substances like chlorhexidine derivatives showed higher effectivity than natural plant compounds, the synthetic agents did not create a eubiotic system. Therefore, it will be interesting to test the effect of plant substances added to dental care products in the eubiosys/dysbiosis of oral tissues.

The combination of different plant compounds could increase their effectiveness in inactivating pathogenic bacteria. The interaction of essential oil components can present various effects; an example is the synergy shown in binary combinations of *C. citratus* (0.44 mg/mL) and *Allium cepa* (5.26 mg/mL) essential oils that at decreased doses (0.22 mg/mL + 1.28 mg/mL) inhibited the growth of *S. aureus* [51]. In addition, ternary combinations of protocatechuic acid (12.98 mM), vanillic acid (11.8 mM), and catechin (13.78 mM), phenolic compounds found in several plants, reduced their effective doses (1.62 mM + 0.74 mM + 0.05 mM), inhibiting the growth of *E. coli* [52]. Therefore, binary or tertiary mixtures of plant compounds can be used to combat pathogenic bacteria; their combination can increase effectiveness, making them an attractive option to replace synthetic compounds. However, more studies testing the effects of these combinations are needed, specifically against cariogenic bacteria. Also, plant essential oils can present antioxidant capacity and anticarcinogenic potential, in contrast to other synthetic antibacterial agents commonly used in oral care products.

### 4.2. Antioxidant Properties

The high incidence of deaths caused by oral cancer has stimulated treatment developments; some are chemotherapy, radiotherapy, and antioxidant agents [61]. Chemotherapy uses cisplatin, 5-fluorouracil, and cetuximab; these are responsible for inhibiting the epidermal growth factor receptor involved in the angiogenesis and metastasis of oral cancer cells [62]. Another example of these agents is YC-1 [3-(5’-hydroxymethyl-2’-furyl)-1-benzylindazole], which can inhibit the protein resistance to multiple drugs and is capable of reducing the content of cancer cells by the stimulus of apoptosis [63]. However, it has been reported that some of these compounds can be cytotoxic and cause diarrhea, nausea, and vomiting [64]. Thus, even when these agents are a useful alternative for oral cancer treatment, they produce dangerous collateral effects; in addition, it is always preferred to prevent the presence of this disease. The high costs of chemotherapy and its adverse side effects have allowed exploring natural plant derivatives to avoid and combat oral cancer [15].

Derivatives of natural plants possess antioxidant and anticarcinogenic properties, which allows them to be used as a preventive measure of oral cancer (Table 3). An example of these compounds is turmeric acid from *Curcuma longa*, polyphenolics from green tea, apigenin from moringa plants, cyanidin from berries, gingerol from ginger roots, eugenol and thymol from essential oils that inhibited several biochemical parameters of cancerous cells, arresting tumor development and causing apoptosis [65,66,67,68,69,70]. Thus, studying natural plant derivatives facilitates understanding how they act to prevent this deadly disease.

**Figure 5:**
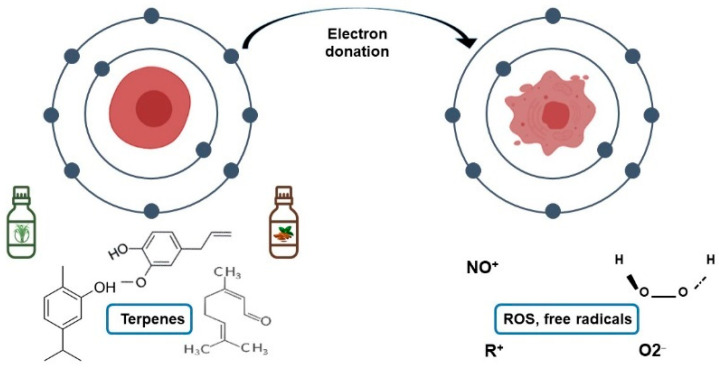
The antioxidant capacity of secondary metabolites in essential oils reduces the oxidative stress of oral tissue produced by free radicals and ROS.

The anticarcinogenic capacity of natural plant derivatives favors their introduction into dental products that could prevent oral cancer. *Menta piperita* extract is an example of compounds that have been introduced to dental products, which has a cytotoxic effect on tumor cell lines. On the other hand, thymol is a derivative of thyme; this terpenoid is considered one of the main antibacterial agents in mouthwashes and an antioxidant compound since it reduces the damage caused by hydrogen peroxide and nitrous oxide, among others ROS (Figure 5) [70]. Current knowledge indicates that bioactive compounds from natural plants can prevent cancer by protecting DNA, stabilizing ROS, and regulating the cell cycle [71]. Therefore, incorporating plant compounds into dental products can prevent oral cancer by propitiating a balanced oxidative status.

### 4.3. Flavoring Properties

The diversity of aroma notes from plant compounds allows them to be added in different matrices (Table 4). Essential oils provide pleasant sensory characteristics to several products [54]. For example, *C. citratus* essential oil is used as a food ingredient, and it contains citral and myrcene as main constituents, providing a fresh flavor and a pleasant citrus smell [14]. The essential oils obtained from M. *bracteata* and *O. basilicum* plants contain flavonoids, such as gallic acid and quercetin that generate sweet and bitter flavors. On the other hand, the essential oil of *C. zeylanicum* has components, such as cinnamaldehyde and eugenol that produce the characteristic smell of cinnamon; this has been used as a flavor and odor enhancer in confectionery products and aroma release [17]. Therefore, correct essential oil choice is fundamental since they will grant specific aromatic, antibacterial, and antioxidant properties.

It has been proven that dental products formulated with synthetic compounds have negative sensory characteristics (Table 5). For example, the addition of chlorhexidine in a mouthwash caused 80% of the panelist’s sensations of burning, dryness, and taste alteration [16]. On the other hand, curcuminoid increased the acceptability of taste, odor, and color of dental products [90]. Similarly, an herbal mouthwash Hiora^®^ containing 5 mg of Pilu (*Salvadora persica*), 10 mg of bibhitaka (*Terminalia bellerica*), 10 mg of nagavalli (*Piper betle*), 1.2 mg of gandhapura taila (*Gaultheria fragantissima*), 0.2 mg of ela (*Elettaria cardamomum*), 1.6 mg of peppermint (*Mentha* spp.), and 0.4 mg of yavani (*Trachyspermum ammi*) provided a satisfying taste and a pleasant strong odor; also, a lower burning and dry sensation were obtained with plant extracts when consumers compared against chlorhexidine mouthwashes (0.2%*w/v*) [16]. Furthermore, mouth-rinses are concentrated aqueous solutions with 10–20% alcohol, which works as a solvent for different substances and decreases the unpleasant taste of surfactants and antibacterial agents [38]. However, alcohol can irritate, causing a burning sensation and dehydration of oral tissues. Therefore, oral care products added with essential oils and their derivatives grant appealing odors and taste without using alcohol.

## 5. Conclusions and Future Trends

Oral diseases are related to the proliferation of pathogenic bacteria, mainly *S. mutans,* and the expression of virulence factors. The main factors impacting bacterial virulence in oral cavities are biofilm formation and the production of ROS. These generate a suitable microenvironment for bacterial development, allowing the catabolism of substrates and the beginning of the teeth’ deterioration and oral cancer. Antibacterial agents added to oral care products help prevent oral diseases. However, the incorporation of chlorhexidine, fluorides, and hydrogen peroxide has adverse effects on these products’ sensory acceptability. The negative effects of synthetic antibacterial compounds and the market trend for using natural products have allowed plant essential oils and their constituents to be considered as ingredients for toothpaste and mouthwashes.

Furthermore, the analyzed information shows that adding plant essential oils in dental products favors the sensory acceptability of dental products compared with synthetic compounds. Another important characteristic is their ability to reduce free radicals and prevent the proliferation of cancer cells. Therefore, the mentioned properties make plant-derived compounds an alternative for developing oral care products that effectively combat pathogenic bacteria, ROS and are acceptable for consumers. Future research is needed to evaluate the effect of plant compounds in dental care products regarding the maintenance of eubiosis in oral tissues. The interaction of different plant substances can be considered in the formulation of dental products to test the synergist effect evaluated in-vitro. Also, incorporating these substances in different oral products is an observed trend; therefore, their effect on dental plaque formation, antioxidant status and consumer acceptability must also be evaluated. Finally, it has to be mentioned that more in-vivo studies are needed to prove the efficacy of oral care products added with plant substances.

## Figures and Tables

**Table 1:** General advantages and disadvantages of the most common antibacterial compounds used in commercial mouthwashes.

Antibacterial Agent	Advantages	Disadvantages	References
Chlorhexidine	Wide antimicrobial spectrum	Alterations in the sense of taste, mucous peeling	[40,41]
Sodium fluoride	Remineralizing properties	Toxic if it is ingested	[12,13]
Cetyl pyridinium chloride	Inhibit bacterial adhesion on surfaces	Staining of teeth	[38]
Hypochlorous acid	It does not cause damage to the environment	Easily degraded	[13]
Hydrogen peroxide	Tooth whitener and antiplaque agent	Produce temporary tooth sensitivity	[44]
Essential oils	They have antimicrobial activity, do not present local side effects, have a variety of aromas and flavors with potential appealing for the formulation of dental care products	Intense taste	[38]

**Table 2:** Minimum inhibitory concentrations (MIC) and bactericides (MBC) of chlorhexidine digluconate, Listerine^®^, essential oils and their derivatives against planktonic cells of *S. mutans*.

Component	MIC (mg/mL)	MBC (mg/mL)	Reference
Menthol	0.5	1.0	[53]
Eugenol	0.1	0.2	[54]
Thymol	0.312	0.312	[55]
Eucalyptol	0.250	0.5	[53]
Methyl salicylate	1.0	1.0	[53]
*Cymbopogon citratus*	0.125–0.250	0.250–0.5	[54]
*Cinnamon zeylanicum*	0.250–0.5	0.5–1	[54]
Fruit extract of *Berberis vulgaris*	0.064	0.128	[56]
Peel extract of *Citrus aurantifolia*	0.02	-	[57]
Bark extract of *Acacia nilotica*	0.3	4	[58]
Tea of *Camellia sinensis*	6.25	12.5	[59]
Tea of *Hibiscus sabdariffa*	25	50	[59]
Listerine^®^	1.25	-	[60]
Chlorhexidine digluconate	0.0094	0.0094	[60]
Chlorhexidine dihydrochloride	0.00092	-	[57]

- Not determined.

**Table 3:** Anticarcinogenic mechanisms of some plant compounds used in dental products.

Component	Proposed Mechanisms	Reference
Turmeric acid from *Curcuma longa*	Inhibits the transcription of NF-kB, Cox-2, TNF-α, cyclin D1, ICAM-1, c-myc, Bcl-2, MMP-9, iNOS, IL-6, IL-8, causes cell cycle arrest, promotes apoptosis, and acts as ROS scavenger.	[65,66]
Epicatechin, epigallocatechin, epicatechin-3-gallate, and epigallocatechin-3-gallate from *Camelia sinensis*	ROS scavengers, inhibit tumor proliferation, induce apoptosis, arrest cells in G0 and G1 phase, downregulate cyclin D1, increase p14 and p16 proteins levels, blocks angiogenesis.	[67,72]
Apigening from *Moringa oleifera*	Induces apoptosis	[68,73]
Cyanidin from fruits and vegetables	Inhibits tumor cell growth, COX-2 gen, MMP, and EFGR expression.	[74,75]
Gingerol from *Zingiber officinale*	Decreases INOs and TNF-α expression and induces apoptosis.	[69,76]
Eugenol and trans-cinnamaldehyde from *Cinnamomum zeylanicum*	ROS scavenger, inhibits the growth of cancerous cells, decreases Bcl-2, Ki67, VEGF, and CD24 expressions and MDA levels.	[77,78]
Thymol from *Thymus vulgaris*	Induces apoptosis and mitochondrial dysfunction in cancerous cells and inhibits the activity of COX-2 and 5LOX.	[70]

**Table 4:** Examples of sensory attributes (odor and flavor) of some essential oils with antibacterial and antioxidant properties.

Essential Oil	Compound	Odor/Flavor	References
*Melaleuca styphelioides*	Tannins	Woody, bitter	[79]
*Uncaria hook*	Furfural	Caramel, astringent	[80]
*Cymbopogon citratus*	Linalool, limonene, β-myrcene	Floral, sweet, herbaceous, citrus,	[14,81]
*Uncaria hook*	Benzaldehyde	Sweet, astringent	[80,82]
*Satureja Hortensis*	Catechin	Odorless, biter	[14,83]
*Thymus vulgaricus*	Carvacrol	Odorless, spicy	[83,84]
*Syzygium aromaticum*	Eugenol, eugenyl acetate, caryophyllene	Sweet, spice, wood	
*Cinnamomum zeylanicum*	Cinnamaldehyde, eugenol, copaene, β-caryophyllene.	Sweet, wood	[85]
*Citrus limon*	d-limonene, γ-terpinene, β-pinene, β-cymene, α-pinene, α-terpineol and α-thujene	Citrus, herbal, terpenic, woody, and floral aroma descriptors	[86]
*Origanum vulgare*	Thymol, γ-terpinene, carvacrol	Spicy, bitter, pungent, astringent and hay flavor	[87,88]
*Lavandula angustifolia*	Linalool, linalool oxides I and II, linalyl acetate, lavandulyl acetate	Floral, herbal and clove-like odors	[89]

**Table 5:** Sensory attributes of dental products with different antimicrobials.

Compound	Observations	References
Fluoride	Bad taste	[42]
Cetyl pyridinium chloride	Dryness and burning sensation	[91]
Hypochlorous acid	Unpleasant taste, dryness, and irritation sensations	[92]
Peroxide oxygen	Bitter taste	[93]
Chlorhexidine	Intense burning sensation and dryness	[16]
Essential oils	Satisfying taste and a pleasant strong odor	[94]
